# Supplementary material for: FABP4 Controls Fat Mass Expandability (Adipocyte Size and Number) through Inhibition of CD36/SR-B2 Signalling
Source: Int J Mol Sci. 2023 Jan 5;24(2):1032. doi: 10.3390/ijms24021032 (PMC9867004; doi:10.3390/ijms24021032)

SUPPLEMENTARY FILES

Suppl Tables S1: Analysis of gene dataset enrichment in mouse FAT/CD36 knock out gene dataset [55]. Significant enrichments are indicated with asterix for z-score confidence levels >95%. The fatty acid dataset has been previously described (Berger et al, 2012 doi: 10.1002/biot.201200188). Analyses of signalling enrichments in fatty acids, adipose stem cells (ASCs), differentiating (dA) and adipose tissue (AT), JAK2 and LKB1/AMP datasets have been previously described [12] [23]; those of extracellular FABP4 and intracellular FABP4 knock out (FABP4) were retrieved from Yamamoto et al, 2016 [50] and Hurley et al, 2012 [55], respectively.

S1A- External stimuli datasets enrichmed in FAT/CD36 knock out dataset

	Genome		CD36 KO mouse			Fatty acids			Adipose stem Cells (ASCs)			Differenciatiing adipocytes (dA)			Adipose Tissue (AT)			FABP4			intracellular FABP4 inhibition		
	Gene number	Frequency (%)	Gene number	Frequency (%)	Significant z-score	Gene number	Frequency (%)	Significant z-score	Gene number	Frequency (%)	Significant z-score	Gene number	Frequency (%)	Significant z-score	Gene number	Frequency (%)	Significant z-score	Gene number	Frequency (%)	Significant z-score	Gene number	Frequency (%)	Significant z-score
**With stimulus**	17349		1005			541			234			1243			204			###			524		
ABL	218	1,26	24	2,39	*	19	3,5	*	2	0,9		50	4,0	*	3	1,5		21	1,7		7	1,3	
Adenylate cyclase	137	0,79	12	1,19	*	13	2,4	*	4	1,7		25	2,0	*	10	4,9	*	15	1,2		8	1,5	*
AKT	1607	9,26	129	12,84	*	74	14	*	27	11,5		186	15,0	*	27	13,2		169	14	*	138	26	*
ARFs	451	2,60	36	3,58	*	32	5,9		12	5,1		80	6,4	*	13	6,4	*	47	3,8	*	10	1,9	
ATM	462	2,66	47	4,68	*	14	2,6		5	2,1		30	2,4		9	4,4		48	3,9	*	161	31	*
AURKA	232	1,34	19	1,89		10	1,8		0	0,0		22	1,8		2	1,0		23	1,9		84	16	*
CASP1	369	2,13	31	3,08	*	14	2,6		1	0,4		29	2,3		9	4,4	*	43	3,5	*	121	23	*
CDKN2C	405	2,33	12	1,19		29	5,4	*	6	2,6		35	2,8		5	2,5		42	3,4	*	78	15	*
COP1	78	0,45	3	0,30		2	0,4		1	0,4		10	0,8		3	1,5	*	8	0,7		3	0,6	
COX-2 (PTGS2)	1387	7,99	131	13,03	*	34	6,3		18	7,7		59	4,7		15	7,4		133	11	*	5	1	
DUSP1	428	2,47	33	3,28	*	17	3,1		2	0,9		25	2,0		5	2,5		47	3,8	*	106	20	*
FGFR1	317	1,83	34	3,38	*	11	2		3	1,3		28	2,3		7	3,4		33	2,7	*	126	24	*
Glucose oxidase	179	1,03	17	1,69	*	10	1,8	*	2	0,9		25	2,0	*	6	2,9	*	14	1,1		14	2,7	
Glycolysis (2DG)	251	1,45	24	2,39	*	17	3,1	*	5	2,1	*	49	3,9	*	8	3,9	*	30	2,4	*	12	2,3	
HDACs	938	5,41	65	6,47	*	50	9,2	*	13	5,6		104	8,4	*	19	9,3	*	83	6,8	*	25	4,8	
ID1	445	2,56	40	3,98	*	18	3,3		5	2,1		36	2,9		9	4,4		36	2,9		103	20	*
IKBKB	347	2,00	36	3,58	*	12	2,2		3	1,3		23	1,9		7	3,4		40	3,3	*	117	22	*
IKBKE	294	1,69	36	3,58	*	10	1,8		4	1,7		24	1,9		6	2,9		27	2,2	*	130	25	*
IKBKG	364	2,10	33	3,28	*	14	2,6		4	1,7		24	1,9		5	2,5		37	3	*	101	19	*
JAK2	243	1,40	33	3,28	*	24	4,4	*	3	1,3		53	4,3		9	4,4	*	46	3,8	*	19	3,6	*
JAK3	871	5,02	81	8,06	*	31	5,7		12	5,1		67	5,4		13	6,4		98	8	*	31	5,9	
Metformin	1285	7,41	150	14,93		68	13	*	14	6,0		183	14,7		26	12,7		7	0,6		18	3,4	
LSD1	509	2,93	49	4,88	*	28	5,2	*	4	1,7		48	3,9		6	2,9		54	4,4	*			
Erk1/2	2153	12,41	183	18,21	*	78	14	*	32	13,7		151	12,1		31	15,2		204	17	*	285	54	*
MAP4K4	495	2,85	48	4,78	*	17	3,1	*										82	6,7	*			*
MKK5/Erk5	30	0,17	4	0,40		2	0,4		0	0,0		13	1,0	*	4	2,0	*	2	0,2		0	0	
NOX1	1252	7,22	87	8,66		65	12	*	17	7,3		203	16,3	*	35	17,2	*	95	7,7		49	9,4	*
P38MAPK	691	3,98	62	6,17	*	21	3,9		6	2,6		66	5,3	*	16	7,8	*	46	3,8		133	25	*
PDGFRB	279	1,61	29	2,89	*	10	1,8		1	0,4		24	1,9		5	2,5		35	2,9	*	104	20	*
Pi3K	704	4,06	70	6,97	*	28	5,2		10	4,3		64	5,1		11	5,4		67	5,5	*	140	27	
N-RAS	455	2,62	31	3,08		22	4,1	*	4	1,7		105	8,4	*	13	6,4	*	25	2		11	2,1	
RRM2B (P53R2)	81	0,47	7	0,70		6	1,1	*	1	0,4		12	1,0	*	4	2,0	*	5	0,4		1	0,2	
Wnt/bCatenin (CTNNB1)	687	3,96	56	5,57	*	33	6,1	*	4	1,7		56	4,5		15	7,4	*	54	4,4		105	20	*

S1B: Comparison of FAT/CD36 knock out and FABP4 gene datasets with intracellular pathway gene datasets

	Genome		CD36 KO mouse			Fatty acids			Adipose stem Cells (ASCs)			Differenciatiing adipocytes (dA)			Adipose Tissue (AT)			FABP4			intracellular FABP4 inhibition		
	Gene number	Frequency (%)	Gene number	Frequency (%)	Significant z-score	Gene number	Frequency (%)	Significant z-score	Gene number	Frequency (%)	Significant z-score	Gene number	Frequency (%)	Significant z-score	Gene number	Frequency (%)	Significant z-score	Gene number	Frequency (%)	Significant z-score	Gene number	Frequency (%)	Significant z-score
**With stimulus**	17349		1005			541			234			1243			204			###			524		
ABL	218	1,26	24	2,39	*	19	3,5	*	2	0,9		50	4,0	*	3	1,5		21	1,7		7	1,3	
Adenylate cyclase	137	0,79	12	1,19	*	13	2,4	*	4	1,7		25	2,0	*	10	4,9	*	15	1,2		8	1,5	*
AKT	1607	9,26	129	12,84	*	74	14	*	27	11,5		186	15,0	*	27	13,2		169	14	*	138	26	*
ARFs	451	2,60	36	3,58	*	32	5,9		12	5,1		80	6,4	*	13	6,4	*	47	3,8	*	10	1,9	
ATM	462	2,66	47	4,68	*	14	2,6		5	2,1		30	2,4		9	4,4		48	3,9	*	161	31	*
AURKA	232	1,34	19	1,89		10	1,8		0	0,0		22	1,8		2	1,0		23	1,9		84	16	*
CASP1	369	2,13	31	3,08	*	14	2,6		1	0,4		29	2,3		9	4,4	*	43	3,5	*	121	23	*
CDKN2C	405	2,33	12	1,19		29	5,4	*	6	2,6		35	2,8		5	2,5		42	3,4	*	78	15	*
COP1	78	0,45	3	0,30		2	0,4		1	0,4		10	0,8		3	1,5	*	8	0,7		3	0,6	
COX-2 (PTGS2)	1387	7,99	131	13,03	*	34	6,3		18	7,7		59	4,7		15	7,4		133	11	*	5	1	
DUSP1	428	2,47	33	3,28	*	17	3,1		2	0,9		25	2,0		5	2,5		47	3,8	*	106	20	*
FGFR1	317	1,83	34	3,38	*	11	2		3	1,3		28	2,3		7	3,4		33	2,7	*	126	24	*
Glucose oxidase	179	1,03	17	1,69	*	10	1,8	*	2	0,9		25	2,0	*	6	2,9	*	14	1,1		14	2,7	
Glycolysis (2DG)	251	1,45	24	2,39	*	17	3,1	*	5	2,1		49	3,9	*	8	3,9	*	30	2,4	*	12	2,3	
HDACs	938	5,41	65	6,47		50	9,2	*	13	5,6		104	8,4	*	19	9,3	*	83	6,8	*	25	4,8	
ID1	445	2,56	40	3,98	*	18	3,3		5	2,1		36	2,9		9	4,4		36	2,9		103	20	*
IKBKB	347	2,00	36	3,58	*	12	2,2		3	1,3		23	1,9		7	3,4		40	3,3	*	117	22	*
IKBKE	294	1,69	36	3,58	*	10	1,8		4	1,7		24	1,9		6	2,9		27	2,2	*	130	25	*
IKBKG	364	2,10	33	3,28	*	14	2,6		4	1,7		24	1,9		5	2,5		37	3	*	101	19	*
JAK2	243	1,40	33	3,28	*	24	4,4	*	3	1,3		53	4,3		9	4,4	*	46	3,8	*	19	3,6	*
JAK3	871	5,02	81	8,06	*	31	5,7		12	5,1		67	5,4		13	6,4		98	8	*	31	5,9	
Metformin	1285	7,41	150	14,93		68	13	*	14	6,0		183	14,7		26	12,7		7	0,6		18	3,4	
LSD1	509	2,93	49	4,88	*	28	5,2	*	4	1,7		48	3,9		6	2,9		54	4,4	*			
Erk1/2	2153	12,41	183	18,21	*	78	14	*	32	13,7		151	12,1		31	15,2		204	17	*	285	54	*
MAP4K4	495	2,85	48	4,78	*	17	3,1	*										82	6,7	*			*
MKK5/Erk5	30	0,17	4	0,40		2	0,4		0	0,0		13	1,0	*	4	2,0	*	2	0,2		0	0	
NOX1	1252	7,22	87	8,66		65	12	*	17	7,3		203	16,3	*	35	17,2	*	95	7,7		49	9,4	*
P38MAPK	691	3,98	62	6,17	*	21	3,9		6	2,6		66	5,3	*	16	7,8	*	46	3,8		133	25	*
PDGFRB	279	1,61	29	2,89	*	10	1,8		1	0,4		24	1,9		5	2,5		35	2,9	*	104	20	*
Pi3K	704	4,06	70	6,97	*	28	5,2		10	4,3		64	5,1		11	5,4		67	5,5	*	140	27	
N-RAS	455	2,62	31	3,08		22	4,1	*	4	1,7		105	8,4	*	13	6,4	*	25	2		11	2,1	
RRM2B (P53R2)	81	0,47	7	0,70		6	1,1	*	1	0,4		12	1,0	*	4	2,0	*	5	0,4		1	0,2	
Wnt/bCatenin (CTNNB1)	687	3,96	56	5,57	*	33	6,1	*	4	1,7		56	4,5		15	7,4	*	54	4,4		105	20	*

S1C: FAT/CD36 knock out and FABP4 comparison with transcriptional regulator gene datasets

	Genome		CD36 KO mouse			Fatty acids			Adipose stem Cells (ASCs)			Differenciatiing adipocytes (dA)			Adipose Tissue (AT)			FABP4			intracellular FABP4 inhibition		
	Gene number	Frequency (%)	Gene number	Frequency (%)	Significant z-score	Gene number	Frequency (%)	Significant z-score	Gene number	Frequency (%)	Significant z-score	Gene number	Frequency (%)	Significant z-score	Gene number	Frequency (%)	Significant z-score	Gene number	Frequency (%)	Significant z-score	Gene number	Frequency (%)	Significant z-score
**Genes with FT**	15049	63,7				531	85,4		237	67,7		1276	79,5		220	84,9		1133	47,1		286	46,7	
**ARFs**	437	2,90	2	1,08		27	5,08	*	12	5,06		81	6,35	*	13	5,91			0,00		10	3,5	
**CEBPD**	758	5,04	12	6,49		63	11,9	*	10	4,22		88	6,9	*	19	8,64	*	78	6,88	*	46	16,1	*
**CEBPG**	329	2,19	10	5,41	*	19	3,58	*	1	0,42		45	3,53	*	9	4,09		44	3,88	*	40	14	*
**CEBPZ**	445	2,96	12	6,49	*	36	6,78	*	6	2,53		45	3,53		14	6,36	*	46	4,06	*	58	20,3	*
**CHREBP HEPG2**	781	5,19	16	8,65		49	9,23	*	13	5,49		95	7,45	*	18	8,18	*	70	6,18	*	15	5,24	
**CREB**	1074	7,14	20	10,81		84	15,8	*	7	2,95		200	15,7	*	35	15,9	*	110	9,71	*	57	19,9	*
**CREBBP**	498	3,31	6	3,24		28	5,27	*	5	2,11		45	3,53		17	7,73	*	71	6,27	*	31	10,8	*
**CREM**	336	2,23	6	3,24		17	3,2		3	1,27		46	3,61	*	5	2,27		36	3,18	*	34	11,9	*
**EAF1 (ELL)**	491	3,26	9	4,86		16	3,01		12	5,06		46	3,61		11	5		68	6,00	*	60	21	*
**EBF1**	142	0,94	3	1,62		5	0,94		2	0,84		16	1,25		0	0		20	1,77	*	3	1,05	
**ERRA**	1163	7,73	16	8,65		85	16	*	31	13,1	*	138	10,8	*	18	8,18		96	8,47	*			
**ESR1, ESR2**	844	5,61	15	8,11		10	1,88		1	0,42		33	2,59		7	3,18		101	8,91	*	1	0,35	
**Ets**	100	0,66	1	0,54		9	1,69	*	0	0		25	1,96	*	5	2,27	*	14	1,24		2	0,7	
**FABP4intra**	286	1,90	8	4,32	*	19	3,58	*	18	7,59		57	4,47		13	5,91		46	4,06	*			
**FOXO1A**	834	5,54	7	3,78		86	16,2	*	10	4,22		142	11,1	*	22	10	*	70	6,18	*	41	14,3	*
**FOXO3A**	915	6,08	16	8,65	no	43	8,1	no	20	8,44	*	76	5,96		15	6,82		98	8,65	*	43	15	*
**FXRA**	967	6,43	24	12,97	*	33	6,21		11	4,64		98	7,68		21	9,55		121	10,68	*	23	8,04	
**HES1**	297	1,97	13	7,03	*	11	2,07		5	2,11		31	2,43		5	2,27		40	3,53	*	45	15,7	*
**HIF1A**	284	1,89	3	1,62		43	8,1	*	2	0,84		57	4,47	*	8	3,64		31	2,74	*	4	1,4	
**HIF2A**	25	0,17		0,00		4	0,75	*	0	0		13	1,02	*	2	0,91	*	3	0,26		1	0,35	*
**HNF4**	1001	6,65	29	15,68	*	6	1,13		19	8,02		175	13,7	*	29	13,2	*	148	13,06	*	27	9,44	
**LXR**	186	1,24	8	4,32	*	29	5,46	*	2	0,84		38	2,98	*	11	5	*	29	2,56	*	8	2,8	*
**MIZ-1**	43	0,29	2	1,08	no	6	1,13	*	2	0,84		10	0,78	*	1	0,45		5	0,44				
**MYC**	789	5,24	9	4,86		79	14,9	*	11	4,64		158	12,4	*	35	15,9	*	56	4,94		7	2,45	
**NFAT**	97	0,64	5	2,70	*	7	1,32		2	0,84		11	0,86	non	3	1,36		8	0,71		3	1,05	
**NFE2L2(NRF2)**	191	1,27	2	1,08		13	2,45	*	1	0,42		62	4,86	*	11	5	*	36	3,18	*	1	0,35	
**NFKB**	378	2,51	7	3,78		43	8,1	*	7	2,95		102	7,99	*	22	10		48	4,24	*	8	2,8	
**PPARA**	114	0,76	4	2,16		29	5,46	*	0	0		36	2,82	*	13	5,91		17	1,50	*	3	1,05	
**PPARD**	100	0,66	3	1,62		23	4,33	*	1	0,42		30	2,35	*	8	3,64		11	0,97		4	1,4	*
**PPARG**	647	4,30	13	7,03		72	13,6	*	7	2,95		147	11,5	*	33	15		53	4,68		22	7,69	
**PPARGC1A**	624	4,15	11	5,95		43	8,1	*	12	5,06		136	10,7	*	21	9,55		120	10,59	*	17	5,94	
**RARs**	559	3,71	17	9,19	*	30	5,65	*	12	5,06		75	5,88	*	15	6,82		67	5,91	*	62	21,7	*
**RB1**	620	4,12	8	4,32		44	8,29	*	7	2,95		45	3,53	*	9	4,09		75	6,62	*	3	1,05	
**RXRs**	371	2,47	10	5,41	*	30	5,65	*	6	2,53		70	5,49	*	12	5,45		39	3,44	*	36	12,6	*
**SMADS**	125	0,83	2	1,08		14	2,64	*	3	1,27		39	3,06	*	8	3,64		15	1,32		1	0,35	
**SP1**	284	1,89	9	4,86	*	14	2,64		3	1,27		32	2,51		5	2,27		35	3,09	*	42	14,7	*
**SREBP1c**	524	3,48	16	8,65	*	62	11,7	*	8	3,38		113	8,86	*	27	12,3		42	3,71		13	4,55	
**SRF**	197	1,31	7	3,78	*	5	0,94		2	0,84		27	2,12	*	7	3,18		30	2,65	*	38	13,3	*
**STATs**	347	2,31	10	5,41	*	20	3,77		5	2,11		54	4,23	*	12	5,45		42	3,71	*	43	15	*
**USF**	509	3,38	13	7,03	*	29	5,46	*	11	4,64		65	5,09	*	18	8,18		74	6,53	*	51	17,8	*

S1D: Transcriptional regulator signalling genedatasets commonly regulated by FABP4 (extracellular) and intracellular FABP4 inhibition (FABP4i), JAK2 and LBK1/AMPK pathways. Only pathways with significant z-score for confidence levels are reported, with frequency in gene dataset (%).

	FABP4	JAK2	LKB1/AMPK	FABP4i	*Target genes*
ARFs (dA)		6,81			*PPARG*
CEBPD (dA, AT)	6,88	10,47	8,2	20,28	
CEBPG (dA)	3,88	6,81		18,18	
CEBPZ (AT)	4,06	6,28		25,17	
ChREBP (dA, AT)	6,18			7,69	
CREB (dA, AT)	9,71	25,65	9,6	24,83	*G9S2, PPARG*
CREBBP (AT)	6,27			13,99	
CREM (dA)	3,18	5,24		14,69	
EAF1 (ELL)	6,00	6,81		25,87	
EBF1	1,77				
ERRA (ASCs, dA)	8,47	12,57			
Ets (dA, AT)		5,24			
FABP4 (ASCs, dA, AT)	4,06	7,33			
FOXO1A (dA, AT)	6,18	10,47	7,91	18,18	
FOXO3A (ASCs)	8,65	12,57	13,53	23,08	
FXRA	10,68	13,09		10,14	
HES1	3,53			20,28	
HIF1A (dA)	2,74				
HIF2A (dA, AT)					
HNF4 (dA, AT)	13,06	14,66	8,78	11,19	
LXR (dA, AT)	2,56			3,50	*CD36*
MYC (dA, AT)			7,63		
NFAT		3,66			
NFE2L2(NRF2) (dA, AT)	3,18	3,66			
NFKB (dA)	4,24	12,57			
PPARA (dA)	**1,50**	**2,62**	**1,44**		***FAT/CD36***
PPARD (dA)			1,44		*CEBPA*
**PPARG (dA)**		**9,42**			***FAT/CD36, CIDEC, FABP4, PPARG***
PPARGC1A (dA)	10,59	21,47	7,91		
RARs (dA)	5,91	7,85			*PPARG*
RXRs (dA)	3,44	8,90			
SP1	3,09				
SRF (dA)	2,65	6,28		16,43	
STATs (dA)				18,88	
USF (dA)	6,53	7,85		22,03	*PPARG*

Suppl Figure S1: Extracellular FABP4 does not affect adipocyte size or lipolytic activity.

S2A-Realtime monitoring of 3T3-L1 adipocytes treated with recombinant FABP4 (FABP4r 20 nG/mL) in culture media containing either low glucose (1 g/L), high glucose (HG, 4.5 g/L) with or without Insulin 0.05 U/mL.

S2B-C Volume of TG/cell according to cell line treatment.

Data are presented as mean values +/-SEM (n=8 biological replicates) without significant differences in independent experiments in (B), or significant differences with different letters in (C), Anova test p-values $p<0.05$.

A

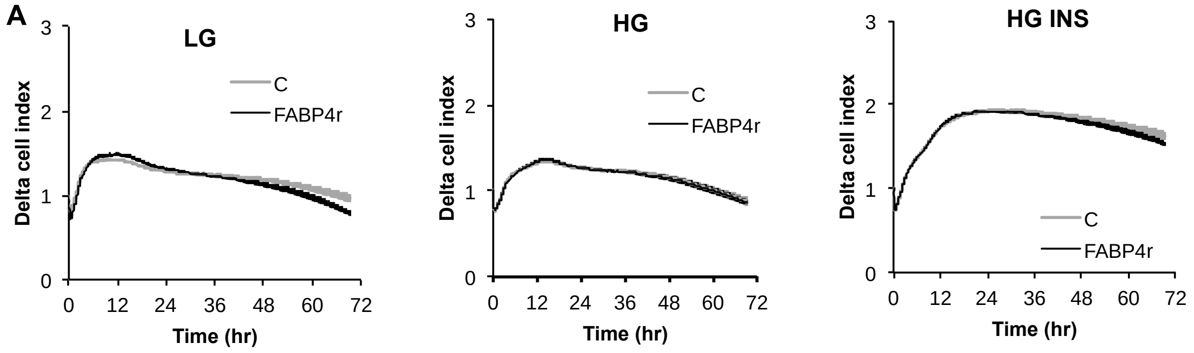

B

**Adipogenesis (3 days)**		
	FABP4r/C	TG/cell (mm3)
3T3L1 adipocytes w/o FCS	LG	1.24 ± 0.13
	HG	1.04 ± 0.06
	HG INS	0.80 ± 0.05
3T3L1 adipocytes with FCS	LG	1.10 ± 0.07
	HG	1.12 ± 0.04
**Basal lipolysis (4h w/o FCS)**		
	FABP4r/C	Adipored intensity/T0
3T3L1 adipocytes	LG	0.98 ± 0.02
	HG	1.03 ± 0.01
	HG INS	0.95 ± 0.01
**Lipid uptake (Oleic acid 10 uM in HG FCS 3 days)**		
	OA/C	TG/cell (mm3)
Human adipocytes	BSA	1.49 ± 0.15
	FABP4r	1.64 ± 0.14

C

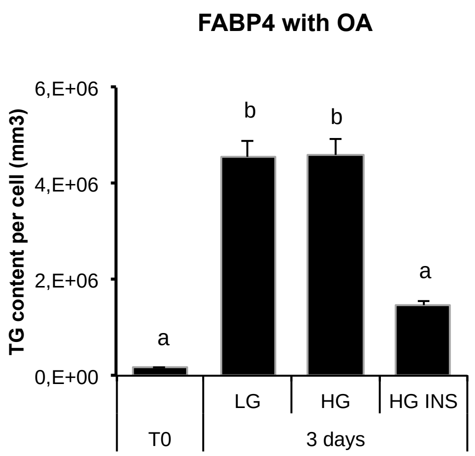

Supplement: Supplementary file 1 [file ijms-24-01032-s001.zip › ijms-2118509-supplementary.pdf]
